# Citric acid: emerging applications of key biotechnology industrial product

**DOI:** 10.1186/s13065-017-0251-y

**Published:** 2017-03-08

**Authors:** Rosaria Ciriminna, Francesco Meneguzzo, Riccardo Delisi, Mario Pagliaro

**Affiliations:** 1 Istituto per lo Studio dei Materiali Nanostrutturati, CNR, via Ugo La Malfa 153, 90146 Palermo, PA Italy; 2 Istituto di Biometeorologia, CNR, via Caproni 8, 50145 Firenze, FI Italy

**Keywords:** Citric acid, Fermentation, White biotechnology, Bioeconomy

## Abstract

Owing to new biotechnological production units mostly located in China, global supply of citric acid in the course of the last two decades rose from less than 0.5 to more than 2 million tonnes becoming the single largest chemical obtained via biomass fermentation and the most widely employed organic acid. Critically reviewing selected research achievements and production trends, we identify the reasons for which this polycarboxylic acid will become a key chemical in the emerging bioeconomy.

**Graphical abstract:**
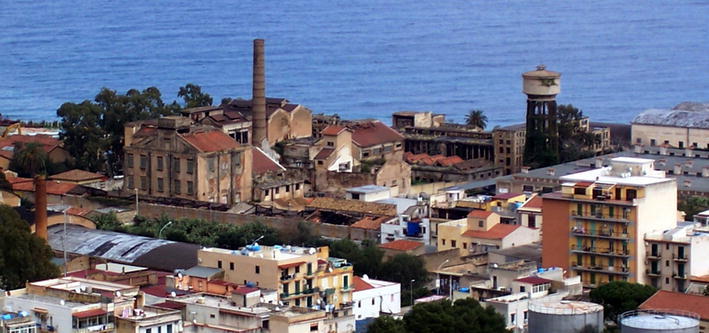
Palermo's Fabbrica Chimica Italiana Goldenberg today. In 1930 it was Europe’s largest citric acid plant (photo courtesy of Aldo Ferrande)

Palermo's Fabbrica Chimica Italiana Goldenberg today. In 1930 it was Europe’s largest citric acid plant (photo courtesy of Aldo Ferrande)

## Background

Citric acid (2-hydroxy-1,2,3-propanetricarboxylic acid, C_6_H_8_O_7_) is an acidulant, preservative, emulsifier, flavorant, sequestrant and buffering agent widely used across many industries especially in food, beverage, pharmaceutical, nutraceutical and cosmetic products [1]. First crystallized from lemon juice and named accordingly by Scheele in Sweden in 1784 [2], citric acid is a tricarboxylic acid whose central role in the metabolism of all aerobic organisms was undisclosed by Krebs in the late 1930s [3].

Owing to its remarkable physico-chemical properties and environmentally benign nature, the use of citric acid across several industrial sectors increased rapidly throughout the 19th century when the acid was directly extracted from concentrated lemon juice, mainly in Sicily (Palermo in 1930 hosted the largest citric acid plant in Europe, Palermo’s Fabbrica Chimica Italiana Goldenberg), by adding lime to precipitate calcium citrate, and then recovering the acid using diluted sulfuric acid.

Along with its elegant chemistry in aqueous and organic solutions, the history of citric acid utilization has been thoroughly recounted by Apelblat in 2014 [4]. In brief, production of citric acid from lemon juice peaked in 1915–1916 at 17,500 tonnes [5], after which it started to decline due to the introduction of the commercial production by sugar fermentation: first in 1919 by *Cytromices* (now known as *Penicillium*) mold in Belgium following the researches of Cappuyns; and then, in 1923, in New York following Currie’s discovery that strains of *Aspergillus niger* (the black mold, a common contaminant of foods belonging to the same family as the penicillins) in acidified solution containing small amounts of inorganic salts afforded unprecedented high yields of the acid (today, 60% on dry matter basis) [6]. Rapidly adopted by numerous other manufacturers, the fermentation process is still used nowadays across the world, and particularly in China, to meet the global demand for the acid, mainly using low cost molasses as raw materials. Interestingly, as recounted by Connor [7], a global citric acid cartel fixed prices for decades. In this study, referring to production, market and recent research achievements, we provide arguments supporting our viewpoint that citric acid will become a key chemical in the emerging bioeconomy [8], with applications beyond conventional usage in the food, pharmaceutical and cosmetic industries.

## Structure, properties and biochemical function

**Fig. 1:**
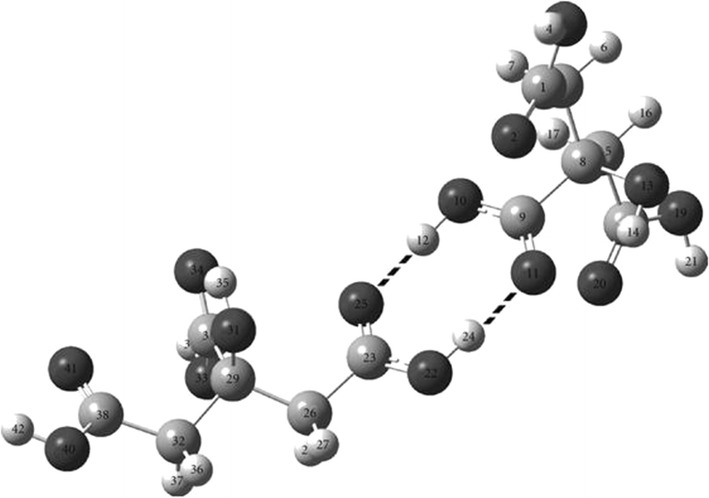
Optimized structure of citric acid dimer from Hartree–Fock ab initio calculations (Adapted from Ref. [12], with kind permission)

The crystalline structure of anhydrous citric acid, obtained by cooling hot concentrated solution of the monohydrate form, was first elucidated by Yuill and Bennett in 1934 by X-ray diffraction [9]. In 1960 Nordman and co-workers further suggested that in the anhydrous form two molecules of the acid are linked through hydrogen bonds between two –COOH groups of each monomer (Fig. 1) [10].

In 1994, Tarakeshwar and Manogaran published the results of the ab initio quantum chemical calculations of electron rich citric acid (and citrate trianion) approximated at the Hartree–Fock level [11]. The team found that citric acid and the citrate trianion have unique features which differentiate them from other α-carboxylic acids. The main difference between the central carboxyl group and the terminal carboxyl groups, highlighted by the ν(C=O) frequencies, was ascribed to an *intramolecular* hydrogen bond between the central hydroxyl hydrogen and one of the terminal carboxyl groups, with the ν(C=O) stretch frequency appearing at a lower frequency than the ν(C=O) stretch of the other terminal carboxyl.

In 2011, Bichara and co-workers published the outcomes of the structural and vibrational theoretical study for the citric acid dimer (Fig. 1) [12]. The values obtained through natural bond orbitals and atoms in molecules calculations, clearly indicate formation of the dimer through hydrogen-bond between two COOH groups of each monomer. Numerous bands of different intensities observed in the vibrational spectra not previously assigned, could now be assigned to the citric acid dimer.

Remarkably, the X-ray analyses of Nordman [10], Glusker and co-workers [13] were undertaken in the context of biochemistry studies. Citric acid, indeed, plays a central role in the biochemical cycle discovered by Krebs in 1937.

**Fig. 2:**
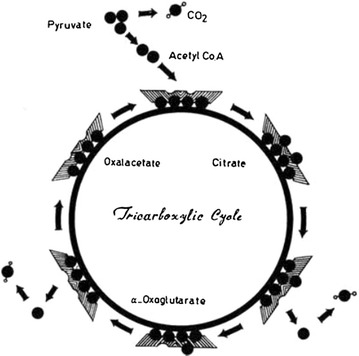
The citric acid cycle devised by Nafissy in 1972, in which the four-carbon skeleton of oxalacetate is a four-wheel carrier to be loaded with two carbon atoms of acetyl group to form the six carbon citrate (Reproduced from Ref. [15], with kind permission)

The citric acid cycle, as lately suggested by Estrada, performs “a kind of concentration” in a self-amplifying cycle in which citrate “pulls in carbon and then it splits, and both parts go back into the cycle, so where you had one you now have two” [14]. Indeed, Fig. 2 reproduced from a 1972 article [15], neatly explains that the sequence of reactions in the Krebs cycle consumes the load of the “carrier” (the four-carbon skeleton of oxalacetate) by transforming it into two molecules of CO_2_, with the unloaded carrier left in oxalacetate form, ready to be loaded again with two-carbon acetyl group.

## Production, properties and applications

Due its eminent biochemical role, it is perhaps not surprising that citric acid is widely distributed in animal species, plants and fruits (Table 1).

**Table 1:** Citric acid in different fruits Reproduced from Ref. [17], with kind permission

Fruit	Citric acid content (mg/100 mL)
Lime	7000
Lemon	5630
Raspberry	2480
Tomato	1018
Pineapple, strawberry, cranberry	200–650

**Fig. 3:**
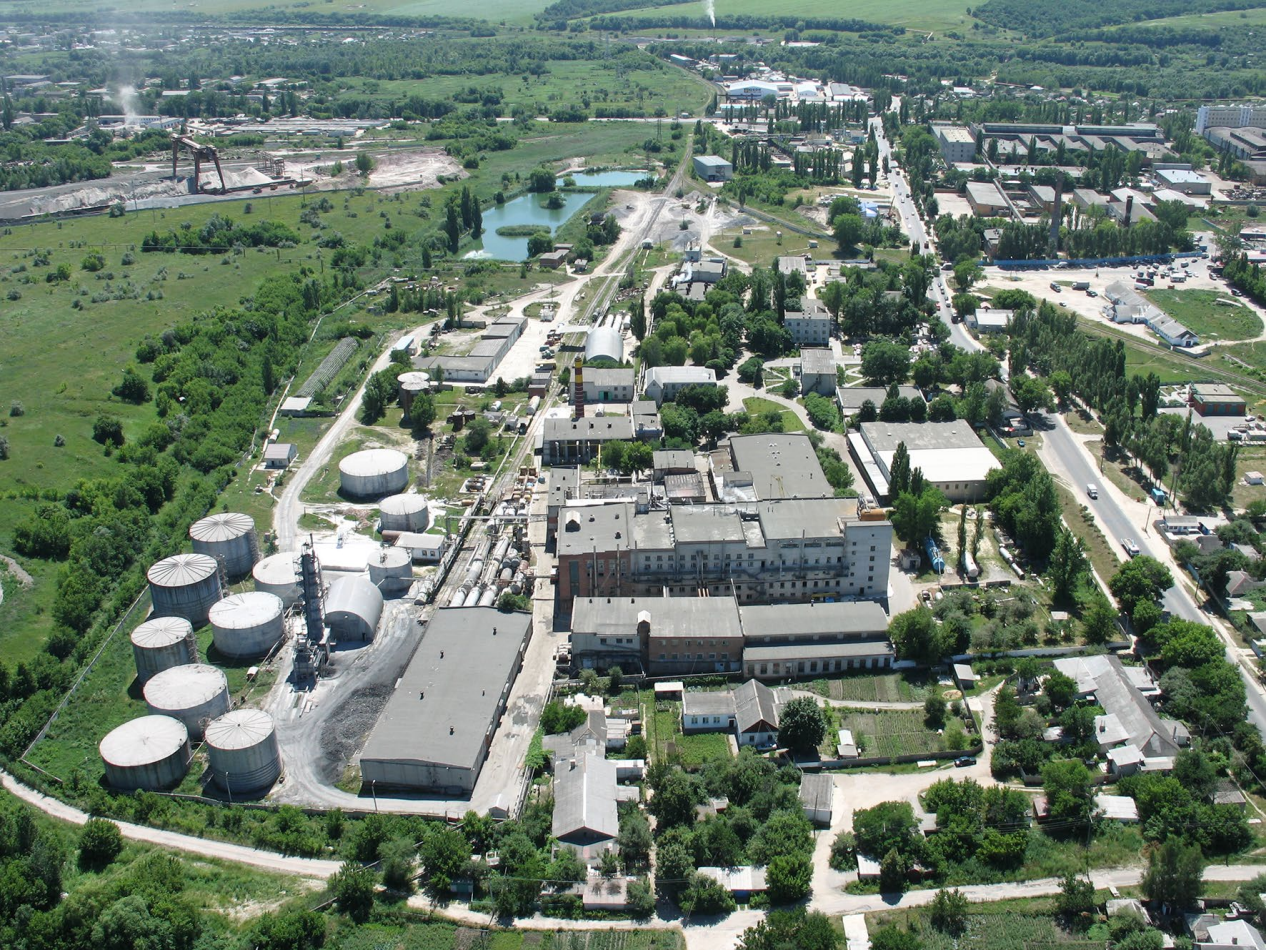
Citric acid plant ‘Citrobel’ in Belgorod (Russia) reproduced from http://www.panoramio.com/photo/29247054, with kind permission

Since the late 1920s, however, the carbohydrate fermentation route has replaced extraction from lemon juice. So efficient and affordable was the new process that as early as of 1934 the acid production cost, using today’s currency values, was €0.2/kg vs. €1.0/kg of 1920 when the acid was still obtained from lemon juice [16]. Today, citric acid is produced at large chemical fermentation plants (Fig. 3) and eventually isolated in two forms, anhydrous and monohydrate. A typical bioreactor is comprised of a batch fermenter (100 m^3^) charged with diluted molasses and minor amounts of inorganic nutrients to which, typically, 5–25 × 10^6^ *A. niger* spores/L are inoculated keeping the reactor under constant stirring (at 50–100 rpm to avoid shear damage on molds). Aeration is supplied to the fermenter by air sparging whereas temperature is kept at 25–27 °C by cooling coils. The production cycle takes from 5 to 8 days depending on the plant, generally affording volumetric yields of 130 kg/m^3^.

To recover the acid from the fermentation broth, a first precipitation with lime is followed by acidification with H_2_SO_4_ and ion exchange, decoloration and crystallisation. The acid is generally sold as a white powder comprised of anyhydrous or monohydrate form typically available in 25 kg paper bags or large (500–1000 kg) bags.

In general, the fermentation process generates twice the volume amount of by-products originating both from the carbohydrate raw material and from the downstream process in the form of a solid sludge (gypsum and organic impurities). All co-products are sold for technical, agricultural and feed applications. The organic part of the molasses, after concentration, is sold as a binding agent for feed. The protein rich mycelium resulting is sold as animal feed, while gypsum is marketed as a filler in cement or in medical applications.

In 2012 Ray and co-workers were noting that the increasing demand required “more efficient fermentation process and genetically modified microorganisms for higher yield and purity” [17]. However, while it is true that numerous citric acid suppliers use molasses from genetically modified corn and genetically modified sugar beet, other manufacturers produce only citrate products certified to originate from carbohydrates obtained from non-genetically modified crops and without any involvement of microorganisms derived from recombinant DNA technology.

Odourless and colourless citric acid is highly soluble in water (62.07% at 25 °C) [18] and slightly hygroscopic. From an environmental viewpoint, the acid quickly degrades in surface waters, and poses no hazards to the environment or to human health [19]. Once dissolved in water, it shows weak acidity but a strongly acid taste which affects sweetness and provides a fruity tartness for which it is widely used to complement fruit flavours in the food and beverage industry. In combination with citrate, the acid shows excellent buffering capacity, while its excellent metal ions chelating properties add to the physico-chemical properties that make it ideally suited for food, cosmetic, nutraceutical and pharmaceutical applications (Table 2), whose number testifies to its exquisite versatility. The acid has the E330 food ingredient code in the European Union (E331 and E332, respectively, for sodium and potassium citrate) indicating a food additive that may be used *quantum satis*. Similarly, it has the GRAS (Generally Recognized as Safe) status in the US. It is somehow ironic that citric acid, once extracted from lemon juice, today is rather added to most lemon, lime or citrus soft drinks at 0.1–0.4% dosage levels. The acid indeed allows to enhance the tangy flavour and to retain quality due to metal ion sequestering properties which help in preventing oxidation that causes flavour and colour loss.

**Table 2:** Main applications of citric acid and related chemical function

Application	Reason
Pharmaceutically active substances, pharmaceuticals, personal care and cosmetic products	Many APIs are supplied as their citrate salt. Effervescent tablets and preparations (via reaction with bicarbonate or carbonate), aiding the dissolution of APIs and improving palatability. Effervescent systems are widely used in teeth-cleaning products, pain relief and vitamin tablets. Very effective buffering system for pH control used in a wide range of for improving stability
Food	Enhancing the activity of antioxidant preservatives (citrate powerful chelating agent for trace metal ions)
Flavouring agent	Sharp, acid taste of citric acid can help mask the unpleasant, medicinal taste of pharmaceuticals
Diuretic	Potassium citrate has diuretic properties
Blood anticoagulant	Citrate chelates calcium, reducing the tendency for blood to clot
Environmental remediation	Chelating agent sequestering heavy metals, including radioactive isotopes, easing also removal of hydrophobic organic compounds
Beverage	Acidulant and pH stabilizer

Compared to the numerous applications identified by Soccol and co-workers in 2006 [20], in the subsequent decade the significant decrease in price and increase in production has opened the route to several new usages of citric acid that had remained idle due to prolonged high prices.

## Emerging uses

Research on new uses and applications of citric acid is currently flourishing, as testified for example by new books published [4], following the still very relevant book written in 1975 by two leading industry’s practitioners [21]. A first noticeable new use is in household detergents and dishwashing cleaners (approximately 13% of the global citric acid market) as a co-builder with zeolites, mainly in concentrated liquid detergents. Citric acid acts as builder, chelating water hardness Ca^2+^ and Mg^2+^ ions but, contrarily to phosphate builders, it does not contribute to the eutrophication of acquatic systems. Since 2017, furthermore, phosphates in dishwasher detergents already banned in the US (since 2010) will be banned in the EU too, leading to increasing consumption of citric acid [22], that will add to increasing use of citrate in domestic cleaners. Numerous other applications will follow. In the following, we provide three examples of recent innovative uses of citric acid that are likely to lead to a further significant market expansion.

### Cross-linker

Citric acid is successfully applied to crosslink many other materials, including ultrafine protein fibers for biomedical applications [23], polyols for making biodegradable films suitable for example for for eco-friendly packaging [24], and with hydroxyapatite to make bioceramic composites for orthopedic tissue engineering [25].

Goyanes and co-workers simply cross-linked citric acid with starch using glycerol as plasticizer by heating a mixture of starch, glycerol, water and citric acid at 75–85 °C. The resulting films with citric acid processed at 75 °C showed a significant decrease in both moisture absorption and water vapor permeability, namely the two main parameters affecting the barrier properties of packaging films. Crosslinking the starch–glycerol films with citric acid, furthermore, significantly improves the poor thermal degradation and mechanical properties of starch films [26].

A significant new application of citric acid as crosslinking agent was discovered in 2011 by Rothenberg and Alberts at the University of Amsterdam, who found that glycerol and citric acid polymerize to form a thermoset resin, soluble in water, showing several important properties including quick degradation in the environment. Until the introduction of this thermoset, nearly all biodegradable plastics have been thermoplastic polymers. Combining citric acid dissolved in glycerol at a temperature above the boiling point of water at ambient pressure and below 130 °C gives a hard polyester resin by a straightforward Fisher esterification process [27]. The boiling points of glycerol (290 °C) and the decomposition temperature of citric acid (175 °C) ensure that water is the only compound liberated as steam, as no decarboxylation takes place at T < 150 °C.

**Fig. 4:**
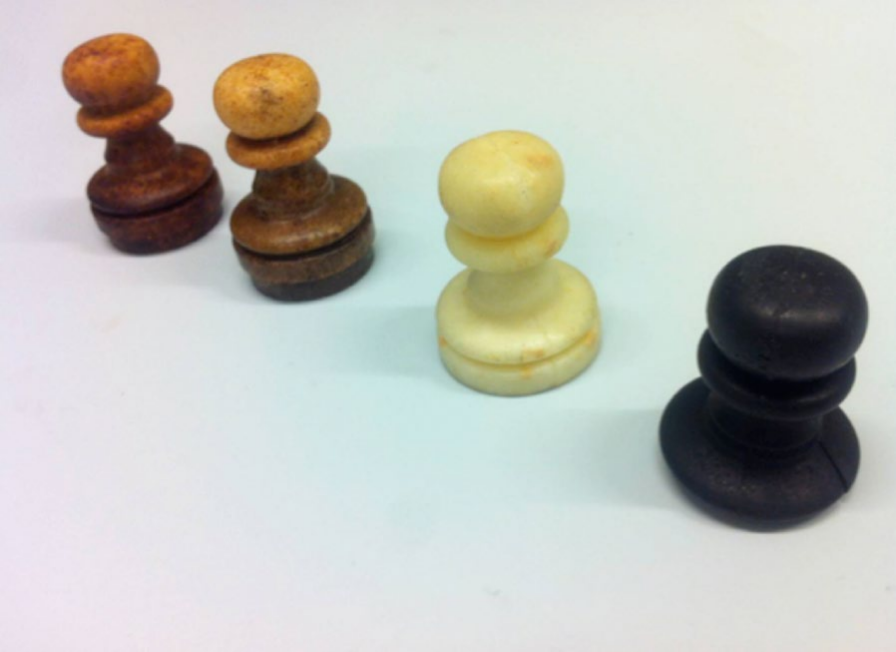
Pawns made of wood next to other samples made of Glycix-GX, the new thermoset resin obtained from citric acid and glycerol (Image courtesy of Professor Gadi Rothenberg)

The resulting polymer is a “bio-bakelite”, a hard three-dimensional polyester which adheres to other materials and can therefore be used in combination with steel, glass, metals and other solid materials used for making inflexible plastic items such as computer and telephone casings, insulation foam, trays, tables and lamps. The extent of crosslinking is controlled by the reaction conditions, most notably temperature, reaction time, and glycerol:citric acid ratio. The higher the extent of crosslinking, the lower the rate of degradation in water. Highly crosslinked samples (Fig. 4) can survive for months in water, and indefinitely in air.

Dubbed “Plantics-GX” by the start-up manufacturing company Plantics, the resin is currently produced on tonne scale at a pilot plant in the Netherlands. The polymer is also inherently safe as it bears no N atom and no S atoms, so there is no possibility of toxic gases during combustion. Full biodegradability ensures that the composite can be disposed of as organic waste as the material hydrolyzes in water making the bio-based particulate available for biological degradation.

### Disinfectant

Citric acid is an excellent, harmless disinfectant against several viruses, including human norovirus. For example, added to norovirus-like particles, citrate precisely binds at the binding pocket on the histo-blood group antigens involved in attaching to host ligands, preventing the transmission of these viruses, as well as reducing symptoms in those already infected with noroviruses [28]. In detail, citrate was also found to bind the norovirus *P* domain, pointing to a broad reactivity among diverse noroviruses. Easily transmitted through contaminated hands or contaminated food, noroviruses cause frequent gastroenteritis outbreaks in community settings such as hospitals, cruise ships, and schools. A commercial paper tissue, containing a middle layer impregnated with citric acid (7.51%) and sodium lauryl sulphate (2.02%), kills the viruses emitted in the form of tiny droplets in the tissue paper after sneezing, coughing or blowing of the nose into the tissue. When moisture hits the middle layer, sodium lauryl sulphate disrupts the lipid envelope of many viruses, whereas citric acid disrupts rhinoviruses, which do not have a lipid envelope, but are sensitive to acids, thereby preventing transfer back to the hands and to surfaces with which the tissue comes into contact [29]. The biocidal product can also be used for the disinfection of surfaces where cold and flu viruses can survive for more than 24 h.

### Environmental remediation

Due to its excellent metal chelating properties, citric acid is widely used to clean industrial sites, including nuclear sites contaminated with radionuclides [30], and soils polluted with heavy metals. For example, not only the citric moiety facilitates the removal of metals in soils [31], but it also enhances the soil desorption of hydrophobic organic compounds from soils [32]. Further enhancing the potential to remove mixed contaminants from soils, recent research in China has shown that when combined with rhamnolipid biosurfactants, citric acid affords unprecedented capacity in soil environmental remediation (better than most thermal or chemical treatments) through biobased chemical agents that are not only environmentally compatible, but also promote soil ecological restoration after remediation [33].

### Extracting agent

**Fig. 5:**
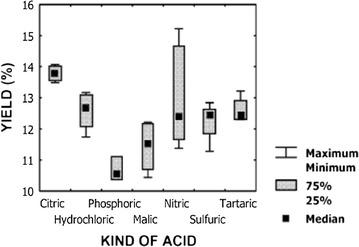
Effect of the nature of acid on pectin extraction yield (Reproduced from Ref. [34], with kind permission)

In 2005, Brazilian researchers first showed that citric acid can be successfully used in place of toxic mineral acids to recover pectin from apple pomace [34]. Pectin extraction yield with citric acid showed the highest average value (13.75%, Fig. 5). Although nitric acid sometimes showed the highest yield, the associated variability was very large, let alone the harmful effluents generated.

Pectin is extracted under reflux in a condensation system at 97 °C (solute/solvent 1:50), using water acidified with citric acid to pH 2.5, and apple flour as raw material. The optimal citric acid concentration is 62 g/L. After 150 min, pectin with excellent degree of esterification (DE = 68.84%) was isolated. Remarkably, the pectin yield was significantly higher using flour as raw material in place of the pomace, as protopectin is more available in small particles than in large ones. Due to its chemical properties and health beneficial effects, the use of pectin is growing across many industrial sectors [35], while its scarcity on the market due to obsolete production processes generating large amounts of waste has recently led to unprecedented high prices.

### Produce preservative

The use of citric acid to reduce microbiological activity, thereby enhancing the stability of concentrates, is well known for example to orange juice makers, who add the acid to concentrates delivered to customers in the beverage industry. Formulated along with other ingredients, citric acid affords an effective commercial antioxidant (NatureSeal), which preserves the aspect (texture and colour) and the organoleptic qualities of several fruits, making them appearing fresh. In tests with fresh-cut apples, for example, the inhibitor out-performs both ascorbic acid (vitamin C) and citric acid when used alone [36].

Another important recent advance is the aqueous solution of citric acid, lactic acid, hydrogen peroxide and a proprietary hydrogen peroxide stabilizer (to slow the decomposition of hydrogen peroxide to water and oxygen gas, Eq. 1), comprising a produce wash (First Step + 10), whose antimicrobial effect is due to the formation of perorganic acids (Eq. 2) [37].

1 $${\text{H}}_{ 2} {\text{O}}_{ 2} \to {\text{H}}_{ 2} {\text{O }} + {\text{ O}}_{ 2}$$

2 $${\text{H}}_{ 2} {\text{O}}_{ 2} + {\text{ R}} - {\text{COOH}} \to {\text{R}} - {\text{COOOH }} + {\text{ H}}_{ 2} {\text{O}}$$

Buffered citric acid makes bacteria membranes more vulnerable to leakage, keeps the wash water within pH 4.0 inhibiting bacterial growth, while the powerful oxidizing agents perorganic acid and hydrogen peroxide quickly penetrate the lipid bilayer membrane providing rapid inactivation of foodborne pathogenic bacteria, including human pathogens such as *Salmonella*, *Listeria monocytogenes* and *Escherichia coli*. After the produce wash is applied to the raw produce and allowed to drain, the constituent ingredients break down into water, oxygen, and organic acids. No toxic compounds are released to the environment. Indeed, in late 2015, the manufacturing company received a positive food-contact substance notification [38].

## Market and bioeconomy aspects

In 1998 the citric acid market was still held by an oligopoly of companies based in North America and western Europe, when one firm in North America and three in Europe were pled guilty of fixing prices and output levels of citric acid in the US and EU from mid-1991 till 1995 [7]. Shortly afterwards, the market oligopoly was disrupted by the entrance of Chinese manufacturers (Table 3) [39].

**Table 3:** Citric acid plant closures until 2010 Reproduced from Ref. [39], with kind permission

Continent	Country	Company	City	Capacity (t)	Year of closure	Feedstock
Europe	France	Jungbunzlauer	Marckolsheim	40,000	2001	Wheat, maize
Europe	Ireland	ADM	Ringaskiddy	40,000	2005	Molasses
Europe	Spain	Ebro	Cortes	5000	1991	Beet molasses
Europe	UK	Tate & Lyle	Selby	25,000	2007	Molasses
America	Mexico	Tate & Lyle	Cuernavaca	22,000	2003	Molasses
America	US	Haarman & Reimer (Bayer)	Elkhardt	40,000	1998	Maize starch
Asia	China	DSM	Wuxi	40,000	2009	Maize starch
Asia	India	Citric India	Mumbai	3000	1997	Cane molasses
Asia	India	Citrugia biochemicals	Surat	6300	2003	Cane molasses
Asia	India	Gujarat State fertiliser & chemicals	Baroda	12,000	2004	Cane molasses
World				233,300		

Put briefly, while in 1989 the world production of citric acid and citrate salts amounted to about 0.5 million tonnes, in 2015 it exceeded 2 million tonnes, with the global market expected to increase at 3.7% annual rate at least until 2020 [40]. In 2015, China accounted for 59% of world production and for 74% of world exports, hosting the largest producers (Table 4). The only new plant not built in China in the course of the last decade is the 12,000 t/a plant in Kermanshah, Iran.

**Table 4:** World’s main citric acid manufacturers and country headquarter

Company	Country headquarter
Gadot Biochemical Industries	Israel
Weifang Ensign Industry	China
Huangshi Xinghua Biochemical	China
RZBC	China
Anhui COFCO Biochemical	China
Cargill	USA
ADM	USA
Citrique Belge	Belgium
Jungbunzlauer	Switzerland
Tate & Lyle	UK

The sudden abundance of the product, with production output almost doubled in the 2004–2013 decade, led to unprecedented low prices that in 2015 bottomed out at $700/t [41]. As in the case of solar photovoltaic modules [42], manufacturers in Europe and in North America were the petitioners in the investigation of anti-dumping duties imposed on products shipped by Chinese companies, lamenting unfair government subsidies and loans to China’s firms. In Europe, for example, the market investigation [43] carried out by the European Commission in 2008 found out that Chinese domestic prices were around 48% lower than those in the EU market. Since June 2008, duties of almost 50% were applied on Chinese citric acid imports.

Commenting on the impact of said tariffs and imposing definitive duties (varying between 15.3 and 42.7%) in early 2015, EU officers were writing that “the Union industry has recovered from the injury caused by the past dumping of Chinese exporting producers” [44]. Yet, in mid-2016, workers in Belgium at one of the few manufacturing sites left in Europe started a blockade [45]. Similar duties exist for example in the US [46], in South Africa and Brazil. In the latter country, on June 2016 antidumping duties of $803.61 and $823.04/t were applied to two Chinese companies found to be violating the provisions determined in 2012, when both were found part of an existent price undertaking [47].

## Outlook and conclusions

Reviewing selected research achievements and market trends, this study provides a critical overview on citric acid. Obtained from molasses via fermentation on black mold, with its 2 million tonnes yearly output, citric acid is the main biotechnology product of the chemical industry and, in our viewpoint, a key chemical of the nascent bioeconomy. The global and strong demand of consumers for naturals, namely for functional products which are beneficial, and not harmful, both to health and to the environment, will continue to drive the demand of citric acid as ingredient in beverage, food, pharmaceutical and cosmetic products. Second, low price and abundance will originate a number of new, large-scale applications of the highly functionalized citric acid molecule, often in combination with other natural products and green chemicals, such as H_2_O_2_ [48], to formulate new preservatives and antioxidants.

The entry into the international market of new China-based manufacturers has reshaped a chemical market which had existed in its oligopoly state for about 80 years since the inception, in the 1920s, of the commercial fermentation process in western Europe and in the US. Likewise to what happened with photovoltaic (PV) modules, wherein tariffs rapidly enforced in the EU and in the US did *not* stop global expansion of solar PV energy to unprecedented levels [49], low price of citric acid boosted its adoption in market segments and world’s regions where it was not traditionally used due to high price, including many south east Asia Pacific countries and Russia, the world’s largest country, which to the best of our knowledge hosts only one citric acid plant (Fig. 3). In conclusion, we argue, existing manufacturers in China will neither reduce production capacity built in the course of the last decade, nor production outputs; but they will rather adapt to prolonged low prices, by increasing the efficiency of the production process. The cost of the raw materials (molasses, *A. niger* water and sulfuric acid) is low and their availability practically unlimited. Under these industrial and market circumstances, developing environmentally friendly chemical technologies based on this eminent green chemical is an important task for today’s chemistry and biotechnology scholars engaged in contemporary sustainable chemistry and green technology research.
